# Involvement of *Mitanins* (female health volunteers) in active malaria surveillance, determinants and challenges in tribal populated malaria endemic villages of Chhattisgarh, India

**DOI:** 10.1186/s12889-017-4565-4

**Published:** 2017-07-11

**Authors:** Mehul Kumar Chourasia, Kamaraju Raghavendra, Rajendra Mohan Bhatt, Dipak Kumar Swain, G. D. P. Dutta, Immo Kleinschmidt

**Affiliations:** 1 National Institute of Malaria Research (ICMR) IIR-WHO Project, Field Unit, Kondagaon, Chhattisgarh India; 2 National Institute of Malaria Research (ICMR), Sector-8, Dwarka, New Delhi, 110077 India; 3 National Institute of Malaria Research (ICMR), Field Unit, Lalpur, Raipur, Chhattisgarh India; 4 Department of Infectious Disease Epidemiology, London School of Hygiene and Tropical Medicine, London, UK

**Keywords:** Mitanin, Asha, Active malaria surveillance, Chhattisgarh, India

## Abstract

**Background:**

Accredited Social Health Activists (ASHA), female health volunteers working at village level have become an integral component of National Health Mission (NHM) in India in the past two decades. *Mitanin* (meaning female friend in local dialect), a precursor of ASHA, play an indispensable role in early detection of health related problems and are helping in improving overall community health status in Chhattisgarh state. The current study was carried out to evaluate the feasibility of involving *Mitanin* in active malaria surveillance work in 80 tribal villages of Chhattisgarh and to explore the challenges and determinants to perform malaria surveillance activities by the *Mitanins*.

**Methods:**

A total of 162 Mitanins were selected and divided into two age and village matched groups. The first group (training plus) of *Mitanins* were given additional training in malaria surveillance activities in whilst the second (standard) group received routine training. All *Mitanins* were interviewed using a structured questionnaire. In-depth interviews were also conducted among randomly selected sub groups of *Mitanins* (five from each group) after the completion of the quantitative survey. Performance of Mitanins was evaluated using pre-defined grading scores (A-E) which included various factors such as educational qualifications and knowledge about malaria, its signs and symptoms and knowledge, attitude and treatment practices.

**Results:**

More number of Mitanins in training plus group has showed better performance (≥ B) than those in the standard group of Mitanins (80% vs 43.5%, *p* = 0.001) after adjusting for socio-demographic factors. Based on the outcome of in-depth interviews, *Mitanin’s* lack of adequate support from supervisors, delayed payment of incentives and lack of appreciation were the major challenges mentioned.

**Conclusion:**

*Mitanins* can play an effective role in active fever surveillance for malaria besides performing other health related tasks at sub-village level after focused education on malaria related activities and proper supervision.

## Background

Community Health Workers (CHW’s) are the key component of the primary health care system in many countries. CHW’s are examples of active community participation towards the betterment of the health of the community. Based on countries’ need and demand it may differ in the levels of health care system within countries, states and regions but in general, their role and responsibility would be similar. This concept of community participation is primarily based on the principle that if members of communities’ make an active effort towards their own and the community’s well-being then they would be more responsive towards primary prevention and radical treatment. This awareness would lead to positive impact on the overall health of the society [1]. With this concept, governments involve community members in the primary health care system. Globally, they have been given a variety of names including Health Auxiliaries, Barefoot Doctors, Shasthyo Shebika, Health Promoters, Health Volunteers, Village Health Workers, Community Health Volunteers and Community Health Workers [2, 3].

The World Health Organization (WHO) describes CHWs as “men or women selected by the society and trained to deal with the ailment of individuals and the community and to work in close relationship with the health care services” [4].

The role of community participation in a range of malaria control activities has a long history in many malaria endemic countries [5, 6]. For example, Latin America’s Volunteer Collaborator Networks (VCNs), unpaid community volunteers, was one of the oldest and successful examples of community participation in malaria control activities. They were trained and supervised by National Malaria Service members [7].

Accredited Social Health Activists (ASHA) and Auxiliary Nurse Midwifery (ANM) are formally trained to support health care system, especially in primary health centers to strengthen basic health services. These village-level community health workers called “ASHA” act as an interface between the community and the public health care system [8].

### Inception of Mitanin and ASHA programme in Chhattisgarh and India [9, 10]

In the early 70’s, Government of India had first introduced the CHW Scheme in the country envisioning “provision of health services at the doorsteps of villager”. However, this scheme was changed over time from CHW in 1977 to Community Health Volunteer in 1980 and Village Health Guides (VHG) in 1981. In 2002, under the ambit of Family Welfare Program, VHG Scheme was completely supported and funded by central government.

To improve access to health care and the health status of rural communities in Chhattisgarh, the state government together with civic society conceptualized and implemented the community health workers (CHWs) programme in 2002. This program was the precursor of ASHA and key reference to developing ASHA program across India. In 2005, CHW programme was merged into National Health Mission (NHM). ASHA workers were then recognized as ‘Mitanin’ in the state which means ‘Female Friend’ in the local dialect. Currently, the state has engaged a total of 69,000 Mitanins. Mitanin Programme is implemented and monitored by Department of Health and Family Welfare, Government of Chhattisgarh in collaboration with State Health Resource Center (SHRC). SHRC is responsible for imparting training to Mitanins in implementing various health programs few of which are incentive based.

### Rationale of the study

ASHA and Mitanin reside in close proximity to the beneficiary’s home, including services delivered at home or to the family and through outreach sessions. It is also expected that Mitanin can play an essential role in detecting problems at the earliest and help in improving overall community health status. In India, several studies have been conducted on various public health care programs related to maternal and child health such as immunization, Integrated Child Development Services, and Janani Suraksha Yojna (Safe Motherhood program) etc. [11–14]. Limited information is available related to ASHA’s and Mitanins’ (Female health volunteers) perception, knowledge and work performance related to malaria.

Multi-country project collaborated by WHO on Implication of Insecticide Resistance (IIR) was carried out in Keshkal, one of the tribal populated sub-districts of Kondagaon district of Chhattisgarh, India aiming to assess impact of insecticide resistance in malaria vectors in presence of Long Lasting Insecticide treated Nets (LLIN) and Indoor Residual spray (IRS) intervention in controlling malaria transmission in malaria endemic area [15]. In this project, Mitanins’ were engaged to carry out surveillance in the selected households in the respective village. Mitanins working under IIR project were classified as ‘IIR Mitanins’. While rest of the Mitanins in the study area were categorized as ‘Non-IIR Mitanins’.

The current study was carried out with the following objectives:

1. To evaluate and compare the knowledge and quality of malaria surveillance work performed by Mitanins’ engaged with IIR- India project in comparison to Mitanins not involved in the project activities (Non-IIR).
2. To explore the effect of various socio-demographics factors such as age and education on malaria surveillance work of the study population
3. To find the various challenges faced by the Mitanins to performance their routine surveillance work related to Malaria

## Methods

### Study area and study design

The present study was carried out in 80 tribal populated villages of Chhattisgarh, India and 162 Mitanins were interviewed during October 2015 to February 2016. This study is a non-blinded randomized controlled trial (additional malaria training being the intervention), with a qualitative component in sub set of population.

### Study arms and subject selection

Mitanins were recruited in two groups. The first group comprise of randomly selected 81 Mitanins (out of 124 Mitanins working under IIR project) with additional malaria training (training plus) and in second groups consist 81 Mitanins (were randomly selected from remaining 300 Non-IIR Mitanins) with routine training (standard). Second group Mitanins were age and villages matched with the group one Mitanins.

### Study intervention

#### Training on malaria surveillance

Two full day basic training sessions on malaria control and surveillance were held in February–March 2015 with groups comprising of 5–15 Mitanins. These Mitanins comprised the training plus group. Basic and field training was imparted in both local dialect (Chhattisgarhi) and in Hindi. Thereafter, these trained IIR Mitanins were engaged for an additional responsibility of malaria fortnight surveillance in their respective villages selected for the IIR project. Their additional responsibility included house to house visit of the cohort children recruited in the IIR-India project. They were trained to collect data on the previous night’s and previous week’s fever history of the children, record temperature (axial), prepare blood slide from fever patients, use bivalent rapid diagnostic tests (RDTs) for detecting *Plasmodium vivax (Pv)* and *P. falciparum (Pf)* and to dispense anti-malarial drugs to malaria positive cases following National Drug Policy. These surveillance activities were regularly monitored by Malaria Surveillance Workers (MSW’s) hired and trained for malaria surveillance among non-cohort population in the IIR project villages and, and supervised by the project staff.

### Study methods

#### Quantitative component:

Structured questionnaire based Interview.

Pre-tested interviewer administered structured questionnaires were used for the study. Study questionnaires were divided into 8 sections; first four sections focused on personal information, duties, selection methods and training, while sections 5–8 included knowledge about malaria, anti-malarial drugs, documentation, slides prepration and use of rapid diagnostic tests (RDTs). Quality of RDT use and blood smear preparation were routinely assessed by laboratory technicians based at the IIR Project site office and improved if needed.

### Evaluation and grading of Mitanins’ performance

Performance of Mitanins was evaluated using pre-defined grading scores (9 point grades) which included various factors such as educational qualifications and knowledge about malaria, including its signs and symptoms and treatment. Nine points score based performance was then converted in to grades from A-E (Table 1).

**Table 1:** Grading factors and scores for Mitanins evaluation

Sl. no	Grading factor	Maximum score	Marking criteria
1	Basic educational qualification	01	Yes – 1, No- 0
2	Level of knowledge about malaria		
2.a	Sign & symptoms of malaria	01	Yes – 1, No- 0
2.b	Knowledge about malaria control	01	Yes – 1, No- 0
3	Slide preparation	01	Yes – 1, No- 0
4	RDT preparation	01	Yes – 1, No- 0
5	Knowledge about anti-malarial drugs		
5.a	Treatment of *Pv*	01	Yes – 1, No- 0
5.b	Treatment of *Pf*	01	Yes – 1, No- 0
6	Form filling and documentation	01	Yes – 1, No- 0
7	Temperature reading	01	Yes – 1, No- 0
Total score		09	
Grade- A 9–8; B 7–6; C 5–4; D 3–2; E 1–0			

### Qualitative component: In-depth interviews

Among all the study participants, purposefully those were selected who can best answer about the given research topic and enhance understanding about the challenges experienced by them. Total ten in-depth interviews (five candidates from each group) were conducted to explore the challenges and difficulties faced by the Mitanins. The topic of the interviews was “day to day challenges in Mitanins’ work”. All interviews were conducted in either Hindi and/or Chhattisgarhi (local dialect) by the trained investigators.

### Statistical analysis

All the study data were double-entered into Epidata- version 3. The cleaned data were analyzed in SPSS version 20.0 (IBM Corp, Armonk, NY). All categorical variables were reported as count and percentages. Continuous variables were reported as mean and standard deviation. ‘Overall Mitanins’ performance’ was the response variable in the analysis. Ordinal variable “Mitanins” performance was converted in to dichotomous variable (less than B grade and ≥ B grade). As observations were paired due to matching between the groups so conditional logistic regressions was chosen. First, univariate conditional logistic regression on Mitanin’s performance in relation to socio demographic determinants such as marital status, type of family, literacy rate and work experience along with Mitanins’ (training plus or standard) was carried out. Then, all factors up to *p* value 0.30 were included in the multivariate conditional logistic regression analysis to investigate the association of socio-demographic factors with Mitanins’ performance after adjusting the effect of other factors.

## Results

A total of 162 Mitanins (81 in each group) were interviewed. Most were married (90.3%), above 35 years of age (61.7%) and staying in nuclear family (52.5%). On an average, Mitanins visited nearly 50 households every week with serving population of ~250. Nearly two third of the Mitanins had primary level of education (69.8%) and literacy rate was 62.3%.

A majority of the respondents said that focus group discussion (70.4%) was held, and half of the study participants (50%) asserted that village meeting were conducted during recruitment of Mitanins in the respective villages. Among all participants, only a few (14.2%) had prior experience of any kind of community level health work. The majority of the Mitanins (51.9%) stated “pressure from family and relatives” as a major reason for joining the Mitanin program compared to “support village health facilities” (22.2%). Both the groups namely the training plus and standard group were comparable, except in literacy (72.8% vs. 50.6%) and education (80.2% vs. 59.2%), in terms of baseline socio-demographic characteristics as shown in Table 2.

**Table 2:** Baseline socio-demographic characteristics of Mitanins (*N* = 162)[IIR(*n* = 81): non IIR(*n* = 81)

Variable	Category	IIR	Non- IIR	Total
		*n* (%)	*n* (%)	*n* (%)
Age	≥ 35 years	31 (38.3)	31 (38.3)	62 (38.3)
Marital status	Married	74 (91.4)	72 (88.9)	146 (90.1)
Family type	Nuclear	38 (46.9)	47 (58)	85 (52.5)
	Extended nuclear	13 (16)	10 (12.3)	23 (14.2)
	Joint	30 (37)	24 (29.6)	54 (33.3)
Education	≥ Primary education	65 (80.2)	48 (59.2)	113 (69.7)
Literacy	Read and write	59 (72.8)	41 (50.6)	100 (61.7)
Household visit /week	Mean (±SD)	52 (±42)	42 (±68)	47 (±57)
Serving population	Mean (±SD)	255 (±134)	206 (±147)	231 (±142)
Focus group discussion held prior to join Mitanin program	Yes	54 (66.7)	60 (74.1)	114 (70.4)
Village meeting held prior to join Mitanin program	Yes	38 (33.3)	43 (53.1)	81 (50)
Prior work experience	Yes	14 (17.3)	09 (11.1)	23 (14.2)
Primary reason of join Mitanin program	Financial incentives	04 (4.9)	04 (4.9)	08 (4.9)
	Opportunity to learn	07 (8.6)	06 (7.4)	13 (8)
	Support village health facilities	23 (28.4)	13 (16)	36 (22.2)
	Social prestige	01 (1.2)	01 (1.2)	02 (1.2)
	Pressure from family and relatives	37 (45.7)	47 (58)	84 (51.9)
	Other	9 (11.1)	10 (12.3)	19 (11.7)

Overall performance of Mitanins’ between the groups.

Table 3 shows the difference in factors and overall performance of Mitanins in both IIR and non- IIR groups. In univariate conditional logistic regression, all individual evaluating factors except slide preparation (*p* = 0.165) and documentation (*p* = 0.122), IIR Mitanins have shown better performance compared with non-IIR Mitanins. In backward conditional logistic regression, educational qualification (*p* = 0.017), knowledge about malaria (0.037), and temperature measurement (*p* < 0.001) were remain as significant factors in the final model.

**Table 3:** Performance of Mitanins in individual factors between the groups

Sl no.	Evaluation factor	IIR (*n* = 81)	Non- IIR (*n* = 81)	Unadjusted *P* value^*^	Adjusted *P* value^#^
1	Basic educational qualification	65 (80.2)	48 (59.3)	*0.003*	*0.017*
2	Level of knowledge about malaria				
2.a	Sign & symptoms of Malaria	78 (96.3)	68 (84)	*0.019*	*0.037*
2.b	Knowledge about Malaria control	80 (98.8)	71 (87.7)	*0.028*	
3.	Slide preparation	80 (98.8)	77 (95.1)	0.165	0.066
4.	RDT preparation	74(91.4)	62 (76.5)	*0.020*	**-**
5.	Knowledge about anti-malarial drugs(*Pv* and *Pf*)	69 (85.2)	51 (63)	*0.002*	**-**
6.	Forms filling & documentation	35 (43.2)	26 (32.1)	0.122	-
7.	Temperature measurement	68 (83.9)	27 (33.3)	*<0.001*	*<0.001*

*Univariate conditional logistic regression

#Backward conditional logistic regression (step 5), *P* value less than 0.05 are statistically significant

### Mitanins performance after adjusting with socio-demographic determinants

Table 4 illustrates the results of univariate and multivariate conditional logistic regression analysis. IIR group Mitanins performance were significantly better than non-IIR group after adjusting for socio-demographic determinants such as age, family type, marital status, literacy rate and prior work experience (*p* < 0.001).

**Table 4:** Mitanins performance after adjusting with socio-demographic determinants (*n* = 162)

Variable	Category	IIR *n* (%)	Non-IIR *n* (%)	Unadjusted Odds ratio (95% CI), *P* value*	Adjusted Odds ratio, (95% CI), *P* value^#^
Overall performance	≥ B	68 (80)	30 (43.5)	6 (2.53–14.24) *<0.001*	4.64 (1.85–11.62) *0.001*
Marital status	Married	74(91.4)	72 (88.9)	1.4 (0.44–4.41) 0.566	**-**
Family type	Nuclear	38 (46.9)	47 (58)	0.61 (0.31–1.18) 0.143	0.76 (0.35–1.67) 0.50
Literate	Yes	59 (72.8)	41 (50.6)	3.57 (1.55–8.26) *0.003*	2.28 (0.87–5.96) 0.095
Prior work experience	Yes	14 (17.3)	09 (11.1)	1.71 (0.68–4.35) 0.257	1.16 (0.38–3.5) 0.795
Purpose of joining Mitanin program	Financial incentives/Learning opportunity/Support health facilities	34 (41.9)	23 (28.3)	1.79 (0.93–3.44) 0.082	1.75 (0.794–3.84) 0.166

*Univariate conditional logistic regression

#Multivariate conditional logistic regression,​ *P* value less than 0.05 are statistically significant

### Major challenges for the Mitanins to perform their work

In the analysis of In-depth interview, five major themes appeared and subsequently placed into framework analysis. It suggests that ‘lack of support from superior staff’, ‘delayed payments’, ‘lack of appreciation’, ‘less incentive’, and ‘intermittent medicine supply’ were major challenges for Mitanins which may affect their routine work (Table 5).

**Table 5:** Summary of major challenges cited by Mitanins during in-depth interview

Sl. no.	List of major challenges	Mitanin									
		IIR					Non-IIR				
		1	2	3	4	5	1	2	3	4	5
1.	Lack of support from superior staff	**√**		**√**	**√**	**√**	**√**		**√**		**√**
2.	Delayed payments by the govt.	**√**	**√**	**√**	**√**	**√**	**√**	**√**	**√**	**√**	**√**
3.	Lack of appreciation and priority treatment of cases referred by them to ANM/Hospital	**√**		**√**			**√**		**√**	**√**	
4.	Less incentive based on performance	**√**	**√**	**√**	**√**	**√**	**√**	**√**	**√**	**√**	**√**
5.	Intermittent medicine supply from the hospital		**√**	**√**	**√**	**√**		**√**	**√**		**√**

## Discussion

ASHA’s and Mitanins are an essential cornerstone of the National Health Mission (NHM) program of India. They are well accepted by the community not only because they are part of them but also due to their accountability to the communities for their health-related activities [1].

### Overall demographic and educational characteristics

An accomplishment of the Mitanin programme largely relies on the individual Mitanin to support for and provide awareness related the meaning and necessity of healthy practices among serving the population. Minimum education level and representation of the local people are key selection criteria if we desire the Mitanin to retain and communicate information to their community members. However, our study showed that nearly 30% of the Mitanins did not have the basic educational qualification. This could affect the NHM program and criteria of basic education. There could be several plausible reasons such as non-availability of literate women in the study villages or reluctance by the young educated girls to join the Mitanin program. Similar findings were reported in a study by Shrivastava et al., 2012 [16]. As per the NHM-ASHA guidelines- 2005, [8] the age of the ASHA’s/Mitanins should be under 45 years. But in our study, we have observed that in some of the villages, age of the Mitanin was over 45 years. This could be a major reason for poor health care awareness in some of the area and may impact on the deliverables of government health care system in the study area.

### Comparative evaluation of Mitanins knowledge and efficiency of malaria surveillance work

Comparative evaluation of both the groups of Mitanins suggested that overall, group one Mitanins (IIR) performance was significantly better than the other group. Study findings imply the rationale of additional and focused training to improve the knowledge and practices related to malaria and surveillance work. Non-IIR group Mitanins can prepare slides as better as IIR Mitanins, but the knowledge about sign, symptoms, and treatment of malaria were significantly lower than the IIR group Mitanins. Apart from being IIR Mitanins, the literacy rate is a significant determinant in unadjusted analysis. This emphasizes the importance of education for female health volunteers for efficient learning and performing the given responsibility of health care of the society.

### Qualitative study findings

There are several key issues such as the financial incentive for community health workers contribute to improved motivation and performance [17]. A number of case studies have shown that regular CHWs are more likely to engage the society in grass-roots health-related issues than the well-trained, but unpaid volunteers [18]. In India, though a nationally prescribed incentive amount an ASHA/Mitanin receives for each activity exists, but varies between the states, and may be many do not actually receive these minimum incentives. Most of the respondents feel that the incentive amount did not meet their expectations. *‘Kabhi Kabhi hum man apan pass k paisa la dethanbimar man la aspatal le jai bar’* (Sometimes we give our own money to take the patients to the hospital) was the response by one of the Mitanin when asked how they bring patients to the hospitals during emergencies. In the year 2009, NHM Evaluation Report submitted by Bajpai et al. [19] suggested a minimal monthly salary in addition to the work incentives. However, to date, it has not been accepted. The report also argued that the financial incentive amount should be increased as it was not commensurate to the quantum of work being performed by the ASHA’s. Based on our findings, we can suggest revamping of financial incentive structure can be considered.

Secondly, many Mitanins asserted that cases referred by them get insufficient attention and they themselves receive insufficient support from the hospital staff which subsequently affects the credibility of their work among the community. Most of the Mitanins stated that they receive the intermittent medicine supply from the Govt. hospitals and is one of major barriers for routine work.

Current study was focused in one of the tribal populated malaria endemic state, Chhattisgarh in India. Results obtained from this study can be generalized but with a caution to other socio- geographic, − cultural and - demographic regions of the country.

These study findings are important for malaria control and elimination programs especially in the context of India that is going for elimination by the year 2030. This can be one of the components that needs immediate attention and also could be relevant to the countries which has similar situation for malaria control with respect to the operations and human resources for health care.

### Study limitations

There are two major constraint of this study. First, as additional training is an intervention, blinding cannot possible from the participants as well as investigators. Secondly, Mitanins of both the groups usually reside in the same village and some time in proximity. Hence, there was chance of “contamination” of nonintervention arm? (i.e., Mitanins who were taught, might then teach Mitanins who were not.)

## Conclusion

Constant training and supervision is backbone of capacity building of Community health workers. Mitanins can also be engaged for active surveillance in malaria endemic areas. Despite general training, knowledge regarding various aspects of Malaria persists. However, different challenges related to operational aspects such as receipt of material in time, delays in payment, due encouragement, multiplicity of activities etc. that hamper the efficiency of the delivery of work needs to be addressed.

## Abbreviations

ANM: Auxiliary Nurse Midwifery
ASHA: Accredited Social Health Activists
CHW’s: Community Health Workers
IIR: Implication of Insecticide Resistance
MSW’s: Malaria Surveillance Workers
NHM: National Health Mission
P*f*: Plasmodium *falciparum*
P*v*: Plasmodium *vivax*
RDT: Rapid diagnostic test
SHRC: State Health Resource Center
VCN’s: Volunteer Collaborator Networks
VHG: Village Health Guides
WHO: World Health Organization
